# Analysis on the Effectiveness and Characteristics of Treatment Modalities for Bowen’s Disease: An Observational Study

**DOI:** 10.3390/jcm11102741

**Published:** 2022-05-12

**Authors:** Hae-Eun Park, Jin-Woo Park, Yeong-Ho Kim, Ju-Hee Han, Chul-Hwan Bang, Young-Min Park, Ji-Hyun Lee

**Affiliations:** Department of Dermatology, Seoul St. Mary’s Hospital, College of Medicine, The Catholic University of Korea, Seoul 06591, Korea; heun1121@naver.com (H.-E.P.); bju127@hanmail.net (J.-W.P.); catesal@naver.com (Y.-H.K.); alwaysmine8@gmail.com (J.-H.H.); mrbangga@catholic.ac.kr (C.-H.B.); 96015367@cmcnu.or.kr (Y.-M.P.)

**Keywords:** Bowen’s disease, cryotherapy, imiquimod, photochemotherapy, therapeutics

## Abstract

Treatment options for Bowen’s disease (BD) include surgical excision, cryotherapy, curettage with cautery, topical 5-fluorouracil or imiquimod, and photodynamic therapy. However, it is not clear which treatment is the most effective due to lack of studies. We reviewed the electronic medical records of 158 patients who were diagnosed with BD and treated at Seoul St. Mary’s Hospital from January 2011 to December 2020. Treatment modalities were surgical excision, cryotherapy, photodynamic therapy, and imiquimod. A total of 121 patients was enrolled in this study. The average treatment period was longest for cryotherapy, followed by imiquimod, PDT, and excision (119.53, 87.75, 68.50, and 1 day, respectively). The therapeutic efficacy was highest in the surgical excision group (100%) and lowest in the PDT group (62.5%). The recurrence rate was highest in the imiquimod group (33.33%). Surprisingly, only in patients treated with cryotherapy, satellite lesions developed in 9.09% of them during follow-up. Surgical excision exhibited the highest clearance rate and the lowest recurrence rate, and its treatment period was the shortest, confirming that it remains the gold standard. In contrast, since cryotherapy demonstrated a relatively high recurrence rate including development of satellite lesions, careful monitoring is required when performing cryotherapy for treatment of BD.

## 1. Introduction

Bowen’s disease (BD) is a cutaneous squamous cell carcinoma in situ that presents as an asymptomatic erythematous scaly plaque with a demarcated margin on sun-exposed areas. The worldwide incidence is not exactly known, and it occurs most commonly in older ages, particularly over 60s. The prognosis of BD is good because the disease grows slowly and responds well to treatment [1]. Bowen’s disease progresses into an invasive squamous cell carcinoma in 3% of the lesions [1]. Treatment options include surgical excision, cryotherapy, curettage with cautery, topical 5-fluorouracil or imiquimod, photodynamic therapy (PDT), and laser therapies.

Surgical excision is usually the most preferred option for a single, small BD because of its efficacy and relatively low costs, but in case of multiple lesions or challenging surgery, non-surgical treatment is another alternative. PDT appears to be safe and effective and has a cosmetic advantage compared to other treatments [2]. These findings allow PDT as an alternative therapy to surgical excision in cases where surgical treatment is difficult. Cryotherapy is a conventional therapy that is relatively less expensive and accessible [1]. Imiquimod 5% cream, approved for actinic keratosis and superficial basal cell carcinoma, is a topical immune modifier that exhibits good therapeutic effects in non-melanoma skin cancers [3,4].

However, it remains uncertain which treatment is the most effective due to lack of controlled trials [1,5]. Evaluation of treatment modalities for BD is difficult considering the potential selection bias to specific forms of treatment in actual practice [5]. In addition, within the same treatment modality, there are variations according to size, location, number of lesions, and treatment regimen among studies, leading to differences of treatment outcomes in actual clinical settings [5].

In this study, we evaluated the effectiveness between treatment modalities of BD in actual clinical practice and analyzed the characteristics of each treatment.

## 2. Materials and Methods

### 2.1. Patients and Enrollment Criteria

We reviewed the electronic medical records of 158 patients who were diagnosed and treated for BD at Seoul St. Mary’s Hospital from January 2011 to December 2020. Inclusion criteria were (1) patients diagnosed with histologically proven BD and (2) patients treated with surgical excision, cryotherapy, PDT, or 5% imiquimod. Exclusion criteria were (1) final non-BD diagnosis after total excision, (2) patients who could not be ‘definitely’ diagnosed histologically with BD despite clinical suspicion, (3) patients later diagnosed with BD through re-biopsy while being treated for actinic keratosis, (4) patients who were treated concurrently with two or more modalities, (5) patients who had already undergone treatments at other clinics before visiting our clinic, and (6) patients with no more than 6 months of follow-up after the last treatment.

A final total of 121 patients was included. This study was approved by the Institutional Review Board of Seoul St. Mary’s Hospital, The Catholic University of Korea (IRB number: #KC21RISI0984).

### 2.2. Definitions of Clearance Rate and Recurrence Rate

When patients exhibited no clinically remaining lesions at the end of each treatment modality, they were determined to be ‘cleared.’ When BD recurred within the follow-up periods, it was classified as ‘recurred.’ The clearance rate indicates the proportion of ‘cleared’ patients among those treated with the modality. The recurrence rate is defined as the proportion of ‘recurred’ among ‘cleared’ patients.

Among the recurred patients, when the newly developed lesion was not located directly at the original site but within 2 cm of its margins, it was defined as a ‘satellite lesion’ [6].

### 2.3. Treatment Protocol

For surgical excision, tumors were excised with a 3~5 mm safety margin depending on site, followed by histological confirmation. For cryotherapy, liquid nitrogen was applied to the BD lesions with timed spot freeze techniques for an average duration of 5–10 s in two or three freeze-thaw cycles. The treatment sessions were performed every 3 or 4 weeks. Patients who had residual lesions after three treatment sessions were classified as not cleared [5]. Imiquimod cream 5% was self-applied once a day, 5 days per week. In PDT, all lesions were treated with a single or two passes of an erbium-yttrium aluminum garnet (Er:YAG) ablative fractional laser before application of topical photosensitizer. Methyl aminolevulinate (MAL) 160 mg/g cream (Metvix^®^; Galderma, Sophia Antipolis, France) was applied to the treatment area (3 mm safety margin around the target lesion) as a topical photosensitizer and covered with an occlusive dressing. After 150–180 min, the area was illuminated with a Waldmann PDT 1200L^®^ at a light dose of 150 J/cm^2^. The interval between treatment sessions was 4 weeks.

Treatment duration for cases other than excision was calculated until the lesions were cleared or the patient changed to another treatment.

## 3. Results

### 3.1. Demographic and Clinical Characteristics of the Patients

Table 1 shows demographic data of the study population and clinical characteristics of the lesions. Among the 121 patients included, 54 (44.63%) were male. The median age was 70 years (39–90 years), and the age group between 61 and 80 years comprised 79 patients (65.29%). Of the total 121 patients, 108 (89.26%) had a solitary lesion, while 7 patients (5.79%) had three or more lesions. More than half of the patients (65.29%) were treated with surgical excision, followed by cryotherapy (24.79%), PDT (6.61%), and imiquimod (3.31%).

When analyzed separately by each treatment modalities, there was no significant difference in the age of the patients between treatment methods (Table A1). Also, the proportion of the solitary lesion was the highest in the surgical excision, which revealed that it is the most suitable treatment option for a single BD.

### 3.2. Treatment Duration and Number of Visits

**Figure 1:**
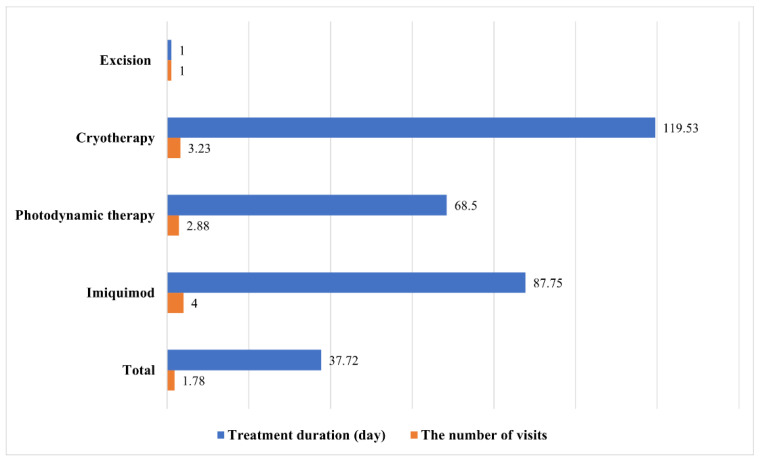
Average treatment duration and number of visits by treatment modality.

As shown in Figure 1, the average treatment period was longest for cryotherapy (119.53 days), followed by imiquimod (87.75 days), PDT (68.5 days), and excision (1 day). In terms of the number of visits for treatment, imiquimod was the highest (4), followed by cryotherapy (3.23), PDT (2.88), and excision (1).

### 3.3. Clearance and Recurrence Rate

**Figure 2:**
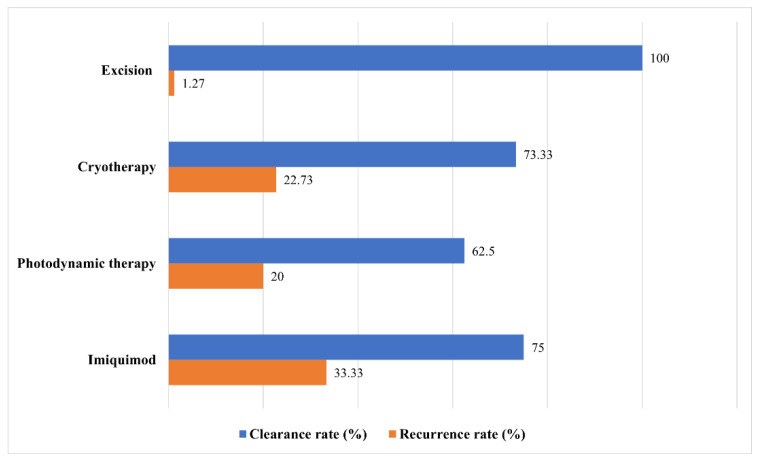
Clearance and recurrence rates by treatment modality.

Figure 2 shows the clearance and recurrence rates of each treatment modality. Surgical excision demonstrated the highest clearance rate and the lowest recurrence rate at 100% and 1.27%, respectively. The clearance rate of cryotherapy was 73.33%, while the recurrence rate was 22.73%. In patients treated with PDT, the clearance rate was 62.5%, and recurrence was noted in 20% of the patients. Imiquimod had the second highest cure rate, but one of three patients whose lesions were cleared experienced relapse.

### 3.4. Development of Satellite Lesions

Among the 22 patients treated with cryotherapy, 2 (9.09%) developed satellite lesions during the follow-up periods (Table 2). In the other treatment groups, there were no satellite lesions.

## 4. Discussion

This study focused on the efficacy and recurrence rate among treatments in actual clinical practice. Excision was the most effective modality in the aspects of treatment and recurrence rate. Cryotherapy demonstrated a poor recurrence rate along with occurrence of satellite lesions. In addition, PDT was not comparable to surgical excision due to the relatively low cure rate and high recurrence rate.

Table 1 shows that it is slightly more common in women (55.37%) than in men (44.63%). Although some previous studies reveal slight male predominance in BD, its incidence was higher in females according to the recent epidemiology of premalignant tumors in Korea [7].

In a retrospective study of 608 patients diagnosed with BD, surgical excision was involved in only 4.9% of treatment failure, which is defined as clinical evidence of residual tumor or tumor recurrence [8]. In addition, a retrospective study of 65 patients reported the recurrence rate of surgical excision as 4.5% [9]. In our study, 65.29% of the patients were treated with surgical excision, which showed the best efficacy of 100% and only 1 case of recurrence, in penile BD. This is consistent with surgical excision as the standard treatment option especially for small, single BD [3] because of its great efficacy and short duration.

Imiquimod is a topical immunomodulatory heterocyclic imidazoquinoline amide with both anti-HPV and antitumor effects and is potentially useful for HPV-associated BD as well as for non-HPV-associated BD [5]. A double-blind randomized controlled trial of 31 patients to evaluate the efficacy of imiquimod 5% cream (once daily for 16 weeks) revealed an efficacy rate of 73% and no relapse during the 9-month follow-up period [10]. In a phase Ⅱ, open-label study, 14 of the 15 patients (93%) exhibited biopsy-proven resolution after 6 weeks of treatment [11]. Another study revealed complete clinical response and recurrence rates of imiquimod cream 5% as 96.8% and 0%, respectively [4]. In our study, of the four patients treated with imiquimod, three obtained complete resolution. Although the sample size is too small to compare with the previously reported cure rates, we assume that the cause of the discrepancy in clearance and recurrence rates between the clinical studies and the actual environment is the premature application method where patients applied the cream themselves. The average age of the imiquimod group is 69.25 years, indicating that this drug is mainly used in the elderly. Therefore, despite education on the application method, compliance remains low. Due to the nature of self-applying rather than being forcibly applied, administration can be easily stopped even with slight discomfort such as pain, pruritus, and erythema. In our study, one failed case was a 66-year-old woman with a lesion on her cheek, who was switched to another treatment because of poor compliance as a result of slight pain and erythema induced by imiquimod. Moreover, there is less monitoring in actual clinical practice compared to a controlled clinical trial. If there are multiple lesions or visiting the hospital is difficult, imiquimod can be suggested as a treatment alternative, but strict evaluation of compliance is required.

PDT is effective for non-surgical patients who have extensive or multiple lesions or for patients who have lesions on poor healing sites or on functional locations such as digital and penile lesions [12]. Complete response rates of 52–100% are reported for PDT on BD at the 3-month follow-up, and recurrence rates ranged from 0 to 46% at the 12-month follow-up [2]. In this study, of the eight patients treated with PDT, five achieved complete remission, one of whom experienced relapse. This low clearance rate of PDT compared to previous studies is presumed to originate primarily from the cost of therapy. Even if treatment of PDT is considered as incomplete clearance following four sessions [2], patients often change to another treatment option after just two or three sessions because of cost issues, resulting in poor compliance. In this study, among the three patients who failed with treatment, one switched to another treatment before completing four sessions. Moreover, the PDT procedure involving type and method of laser used before applying photosensitizer differs by study. Finally, the clinical characteristics of the tumors such as size, number, and location might have contributed to the treatment outcomes as PDT is mainly selected in multiple or bigger lesions where surgical excision is difficult to perform. Surprisingly, the failed cases were all solitary rather than multiple and just located on typical areas like face. Even if the aspects like number and location are excluded, the size of tumor, which was lacking in our data, could be considered as one of the factors leading to treatment failures.

Clearance and recurrence rates of cryotherapy vary by study because of different freezing times, numbers of freeze–thaw cycles (FTC), and characteristics of the lesions [3]. The approximate clearance rate of cryotherapy is reported to be 50–100% [3]. In a comparative study between cryotherapy and PDT, the clearance and recurrence rates were 50% and 10% using one 20 s FTC, respectively [13]. In another comparative study using two 5–10 s FTC, only two lesions did not clear completely, while 13 of the 36 total lesions recurred [14]. Our results did not differ much from those of previous studies. The reason for our relatively low clearance rate compared to a previous study that used a similar treatment regimen [14] was that the procedure was mainly performed by an inexperienced operator such as residents who were still being trained. Nevertheless, cryotherapy remains one of the main treatment options because of its advantages in terms of accessibility and price in cases where it is difficult to perform surgery.

Surprisingly, satellite lesions occurred only when treated with cryotherapy. This can be partially explained by the concept of ‘field cancerization,’ which was first proposed by Slaughter and his colleagues in 1953 to explain the development of multifocal tumors [15]. They observed that clinically normal-looking cells surrounding malignant cells were histologically abnormal and had the potential to develop into a new tumor as local recurrence [15]. This concept has been expanded to include areas that do not exhibit abnormal clinical findings but possess pre-neoplastic molecular changes with microsatellite alterations such as genetic mutations. Sharfaei et al. demonstrated that topical MAL followed by light exposure significantly delayed the development of UV-induced skin tumors [16]. In addition, PDT with ALA (5-aminolevulinic acid) prevented the formation of new non-melanoma skin cancers in patients with field changes [17]. These previous studies proposed that PDT would be an appropriate treatment option to avoid local recurrences including satellite lesions [18]. Moreover, except for cryotherapy, excision, PDT, and imiquimod involve treatment at a constant depth regardless of the area of the lesion. In contrast, since cryotherapy treats the tumor through ice formation, the treatment depth is shallower with distance from the tumor center, leading to weakening of therapeutic intensity [19]. We presume that this leads to development of satellite lesions only in cryotherapy.

There are several limitations in our study. First, as a single-centered study, the sample size of this study was not big enough to analyze by treatment modality. The numbers of cases for PDT and imiquimod were too small to assess and compare the efficacies. Second, the short follow-up period might not have properly captured recurrence and underdiagnosed relapse. Third, there can be a selection bias because non-surgical treatment methods are usually selected when surgical therapy is challenging. Sufficient adjustments were not performed in our study due to lack of clinical data on the characteristics of the tumors such as size. Large-scale studies are necessary to assess treatments at a more meaningful level.

This study focused on the effectiveness of clinical treatment options for BD treatment. We demonstrated that surgical excision provides excellent management for removal of BD if it is possible. However, PDT can be also considered as an effective alternative if patient compliance is ensured.

## Figures and Tables

**Table 1:** Demographic and clinical characteristics of the patients.

Characteristics	Number of Patients (%)
Sex	
Male	54 (44.63)
Female	67 (55.37)
Age (years)	70 (39–90) *
0–20	0 (0)
21–40	1 (0.83)
41–60	23 (19.01)
61–80	79 (65.29)
81–100	18 (14.88)
Number of lesions	
1	108 (89.26)
2	6 (4.96)
3–6	4 (3.31)
≥7	3 (2.48)
Treatment modalities	
Excision	79 (65.29)
Cryotherapy	30 (24.79)
Photodynamic therapy	8 (6.61)
Imiquimod	4 (3.31)

Data are presented as number (%) or median (range) *.

**Table 2:** Development of satellite lesions.

Modality	Development of Satellite Lesions * (%)
Excision	0
Cryotherapy	9.09
Photodynamic therapy	0
Imiquimod	0

* Satellite lesions: those located within 2 cm from the edges of the original lesion.

## Data Availability

Data is contained within the article.

## Appendix A

**Table A1:** Demographic and clinical characteristics of the patients by treatment modalities.

	Excision	Cryotherapy	Photodynamic Therapy	Imiquimod
Sex				
Male	38 (48.10)	11 (36.67)	4 (50.00)	1 (25.00)
Female	41 (51.90)	19 (63.33)	4 (50.00)	3 (75.00)
Age (years) *****	71 (39–87)	70 (46–90)	70.5 (61–80)	70 (57–80)
Number of lesions				
1	72 (91.14)	27 (90.00)	6 (75.00)	3 (75.00)
2	4 (5.06)	1 (3.33)	1 (12.50)	0 (0)
3–6	2 (2.53)	0 (0)	1 (12.50)	1 (25.00)
≥7	1 (1.27)	2 (6.67)	0 (0)	0 (0)

Data are presented as number (%) or median (range) *.
